# Viral and Cellular Factors Leading to the Loss of CD4 Homeostasis in HIV-1 Viremic Nonprogressors

**DOI:** 10.1128/JVI.01499-21

**Published:** 2022-01-12

**Authors:** Marta Colomer-Lluch, Athina Kilpelainen, María Pernas, Ruth Peña, Dan Ouchi, Esther Jimenez-Moyano, Judith Dalmau, Concepción Casado, Cecilio López-Galíndez, Bonaventura Clotet, Javier Martinez-Picado, Julia G. Prado

**Affiliations:** a IrsiCaixa AIDS Research Institute, Badalona, Spain; b Germans Trias i Pujol Research Institute (IGTP), Badalona, Spain; c Unidad de Virología Molecular, Laboratorio de Referencia e Investigación en Retrovirus, Centro Nacional de Microbiología, Instituto de Salud Carlos III, Majadahonda, Madrid, Spain; d Infectious Diseases Department, Hospital Universitari Germans Trias i Pujol, Badalona, Spain; e University of Vic – Central University of Catalonia (UVic-UCC), Vic, Spain; f Catalan Institution for Research and Advanced Studies (ICREA), Barcelona, Spain; Emory University

**Keywords:** HIV-1, viremic nonprogressors, loss of CD4 homeostasis, replication, coreceptor usage, T-cell activation, T-cell maturational phenotypes, CCR5, CXCR6, human immunodeficiency virus

## Abstract

Human immunodeficiency virus type 1 (HIV-1) viremic nonprogressors (VNPs) represent a very rare HIV-1 extreme phenotype. VNPs are characterized by persistent high plasma viremia and maintenance of CD4^+^ T-cell counts in the absence of treatment. However, the causes of nonpathogenic HIV-1 infection in VNPs remain elusive. Here, we identified for the first time two VNPs who experienced the loss of CD4^+^ homeostasis (LoH) after more than 13 years. We characterized in deep detail viral and host factors associated with the LoH and compared with standard VNPs and healthy controls. The viral factors determined included HIV-1 coreceptor usage and replicative capacity. Changes in CD4^+^ and CD8^+^ T-cell activation, maturational phenotype, and expression of CCR5 and CXCR6 in CD4^+^ T-cells were also evaluated as host-related factors. Consistently, we determined a switch in HIV-1 coreceptor use to CXCR4 concomitant with an increase in replicative capacity at the LoH for the two VNPs. Moreover, we delineated an increase in the frequency of HLA-DR+CD38^+^ CD4^+^ and CD8^+^ T cells and traced the augment of naive T-cells upon polyclonal activation with LoH. Remarkably, very low and stable levels of CCR5 and CXCR6 expression in CD4^+^ T-cells were measured over time. Overall, our results demonstrated HIV-1 evolution toward highly pathogenic CXCR4 strains in the context of very limited and stable expression of CCR5 and CXCR6 in CD4^+^ T cells as potential drivers of LoH in VNPs. These data bring novel insights into the correlates of nonpathogenic HIV-1 infection.

**IMPORTANCE** The mechanism behind nonpathogenic human immunodeficiency virus type 1 (HIV-1) infection remains poorly understood, mainly because of the very low frequency of viremic nonprogressors (VNPs). Here, we report two cases of VNPs who experienced the loss of CD4^+^ T-cell homeostasis (LoH) after more than 13 years of HIV-1 infection. The deep characterization of viral and host factors supports the contribution of viral and host factors to the LoH in VNPs. Thus, HIV-1 evolution toward highly replicative CXCR4 strains together with changes in T-cell activation and maturational phenotypes were found. Moreover, we measured very low and stable levels of CCR5 and CXCR6 in CD4^+^ T-cells over time. These findings support viral evolution toward X4 strains limited by coreceptor expression to control HIV-1 pathogenesis and demonstrate the potential of host-dependent factors, yet to be fully elucidated in VNPs, to control HIV-1 pathogenesis.

## INTRODUCTION

The clinical outcome of human immunodeficiency virus type 1 (HIV-1)-infected individuals is characterized by a wide diversity of the average time to disease progression in the absence of active combined antiretroviral therapy (cART). Strikingly, a very small fraction of infected individuals display natural control of HIV-1 replication and pathogenesis.

In this line, a rare subset of individuals termed HIV-1 viremic nonprogressors (VNPs) can preserve CD4^+^ T-cell counts despite high plasma viremia remaining with low immune activation and asymptomatic for many years (1–4). Interestingly, VNPs resemble the simian immunodeficiency virus (SIV) infection in its natural hosts, the sooty mangabeys and the African green monkeys, which exhibit persistent high plasma viral loads (VLs), CD4^+^ T-cell preservation, and low levels of immune activation, resulting in a nonpathogenic viral infection (5–7). In VNPs, the lack of viral pathogenesis has been associated with the preservation of central memory and stem-cell memory CD4^+^ T cells through reduced HIV-1 infection and maintenance of proliferative capacity (3). The preservation of CD4^+^ T-cell central memory cells in VNPs has been associated with thymic repopulation of the naive compartment to maintain T-cell homeostasis despite high viremia (8).

Another subset of individuals that are rare but widely studied are the so-called HIV-1 elite controllers (ECs). ECs spontaneously control viral replication to undetectable levels in plasma. Several studies have reported the loss of virological and immunological control over time in a fraction of ECs (9–14) and comprehensively analyzed the contribution of host and viral factors. Low polyfunctionality of HIV-1 Gag-specific T-cell responses, high viral diversity, and elevated levels of proinflammatory cytokines were observed before the loss of control (10). Additionally, disturbances of T-cell homeostasis, particularly in CD8^+^ T-cells, were found in ECs experiencing loss of immunological control (14). Recently, an increase of CD8^+^ T-cell activation together with a switch in viral tropism preceded the loss of control in a subset of HIV-1 controllers (11).

In contrast to what is known about ECs, it is currently unknown whether the VNPs status will last indefinitely during HIV-1 infection or whether a proportion of VNPs will experience immunological progression in the form of loss of CD4^+^ homeostasis (LoH). The absence of reports of LoH in VNPs might be involved with the rare frequency of the phenotype (<0.1%) (1, 3, 15), the scarce clinical follow-up, and the lack of biological samples associated with LoH. In this study, we identified two cases of VNPs who, after more than 13 years of nonpathogenic HIV-1 infection, eventually experienced LoH. For the first time to our knowledge, we comprehensively analyzed the contribution of viral and host factors to the loss of CD4^+^ homeostasis in VNPs. This information may bring new insights into this extreme phenotype critical to understanding the mechanisms underlining nonpathogenic HIV-1 infection.

## RESULTS

### Clinical characteristics and immunogenetics in VNPs presenting LoH.

**FIG 1:**
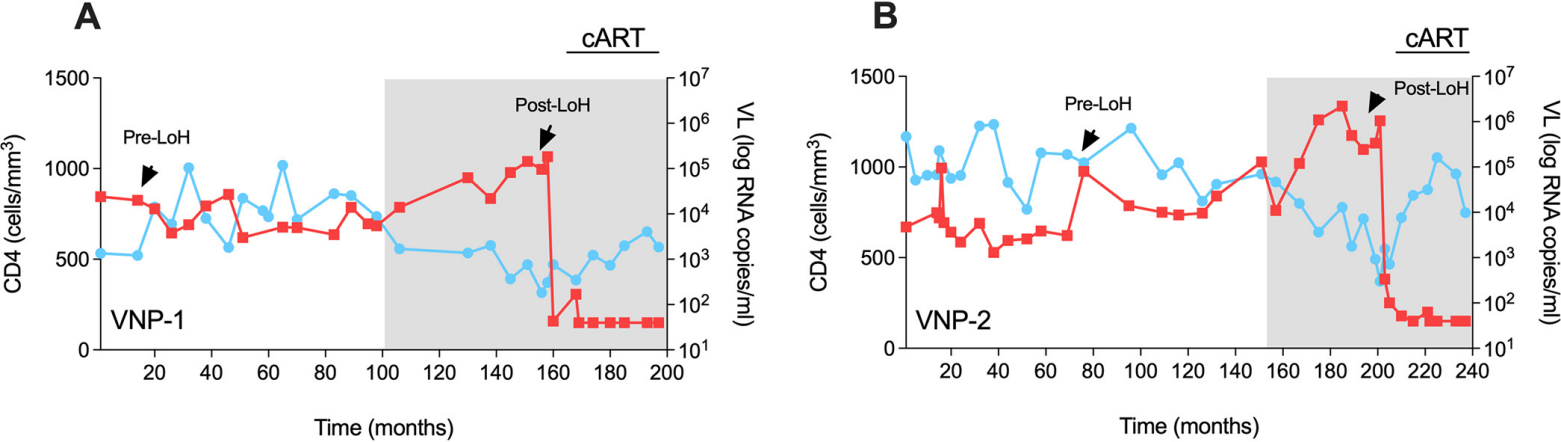
Clinical data of study subjects. Graphs display the evolution of the absolute CD4^+^ T-cell counts as the number of cells/mm^3^ (blue circles) and the HIV-1 viral loads as log RNA copies/mL in plasma (red squares) for VNP-1 (A) and VNP-2 (B) individually over time. Pre- and post-LoH indicate the sampling points by arrows, and the gray-shaded areas depict the period of LoH and cART introduction. VL, viral load; cART, active combined antiretroviral therapy; LoH, loss of CD4^+^ homeostasis; VNP, viremic nonprogressor.

**FIG 2:**
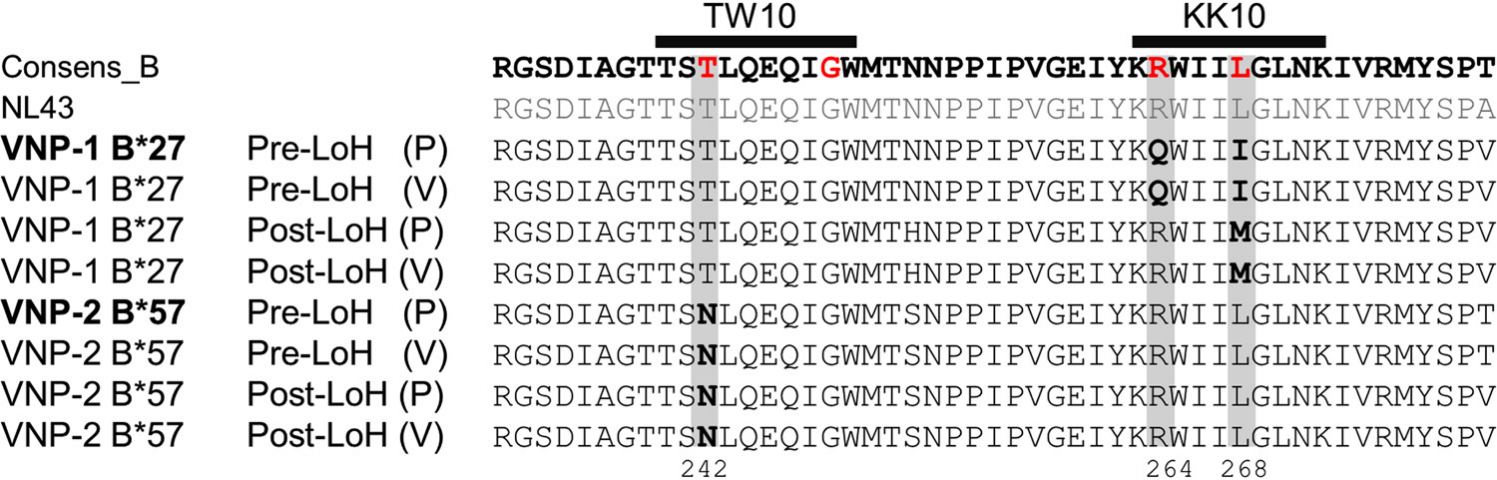
Presence of viral immune escape mutations in HIV-1 Gag sequences of VNPs. The Gag-protease region of the HIV-1 genome from the viral isolates (V) and plasma (P) was sequenced in retrospective pre- and post-LoH samples of the two VNP individuals. Amino acid sequences corresponding to positions 232 to 280 of HIV-1 Gag (relative to an HIV-1 subtype B consensus sequence, as well as laboratory strain NL43) are shown. The presence of escape mutations in the TW10 and KK10 epitopes of HIV-1 gag is highlighted in bold (T242N in TW10 and T264Q and L268I/M in KK10, respectively).

We identified two cases of VNPs (named VNP-1 and VNP-2) presenting LoH from the VNPs cohort established at IrsiCaixa (Badalona, Spain). VNPs were defined as antiretroviral therapy (cART)-naive HIV-1-infected subjects with more than 8 years of follow-up with median absolute CD4^+^ T-cell counts of >500 cells/mm^3^ and sustained plasma viremia of >10^4^ copies/ml without clinical symptoms. This definition in agreement with previous studies of VNPs (16, 17). In our study, both VNP-1 and VNP-2 were cART-naive for 17.8 years and maintained CD4^+^ T-cell homeostasis for 13.4 and 15.5 years, respectively, for VNP-1 and VNP-2. The clinical characteristics and evolution are summarized in Table 1 and Fig. 1. The median viral load (VL; log copies/ml) was 4.11 for VNP-1 and 3.99 for VNP-2. The CD4^+^ T-cell counts (cell/mm^3^) remain stable in both VNPs over the follow-up, with medians of 730.5 for VNP-1 and 954.5 for VNP-2. the Loss of CD4^+^ homeostasis (LoH) control in these two cases was defined by a decrease of 30% in the total CD4^+^ T-cell counts and a rise in viral load (>0.5 log VL) within 1 year while cART-naive (Fig. 1). At the LoH, the median absolute CD4^+^ T-cell counts declined to 382 cells for VNP-1 and 527.5 cells for VNP-2, with a concomitant increase in the median VL (log) to 5.08 and 5.62, respectively. The cART was initiated according to the clinical guidelines at that time (CD4^+^ T-cell counts of ≤350 cells/mm^3^). No evidence of other infections or pathologies was observed coinciding with the LoH. Moreover, we performed high-resolution HLA class I typing on both VNPs as HIV-1 control has been linked to specific HLA class I alleles (18). We found the presence of protective alleles: HLA-B*27:05 in VNP-1 and HLA-B*57:01 in VNP-2 (Table 1). Interestingly, HIV-1 Gag sequences obtained from plasma and viral isolates in both cases pre- and post-LoH demonstrated the preexistence of escape mutations at immunodominant KK10 and TW10 Gag epitopes restricted by B*27:05 and B*57:01, respectively, in VNP-1 and VNP-2 (Fig. 2). The presence of CD8^+^ escape mutations at positions R264Q/L268M in KK10 for VNP-1 and T242N in TW10 in VNP-2 before LoH has not been previously reported and speaks against the contribution of CD8^+^ T-cell responses as a central component of the VNP phenotype in agreement with previous studies (19, 20).

**TABLE 1:** Clinical and Virological Parameters of the VNPs in the Study

	VNP-1	VNP-2
Year of HIV-1 diagnosis	1995	1995
Sex (M/F)	M	M
Time cART naïve (yr)^*a*^	17.8	17.8
Time under virological suppression (yr)	13.4	15.5
CD4^+^ T-cell count/μl at pre-LoH (median)	730.5 [563.3 to 840.8]	954.5 [808.8 to 1,072]
CD4^+^ T-cell count/μl at post-LoH (median)	382 [330 to 450.5]	527.5 [400.5 to 677]
Viral load, log copies RNA/mL at pre-LoH (median)	4.11 [3.71 to 4.32]	3.99 [3.59 to 5.01]
Viral load, log copies RNA/mL at post-LoH (median)	5.08 [4.93 to 5.24]	5.62 [5.43 to 5.94]
HLA class I molecules (A, B, and C alleles)		
A*	01:01/01:01	01:01/02:01
B*	27:05/49:01	57:01/51:01
Cw*	02:02/06:02	01:02/06:02
Time pre-LoH sample (yr)	2001	2002
Time post-LoH sample (yr)	2012	2012

a Abbreviations: cART, combined antiretroviral therapy; IQR, interquartile range; M, male; F, female; LoH, loss of CD4^+^ homeostasis; VNP, viremic nonprogressor.

### Emergence of CXCR4 viral strains with high replicative capacity associate with LoH in VNPs.

First, to investigate the contribution of specific viral traits to LoH in these two VNPs, we examined virus coreceptor usage and replicative capacity as factors widely associated with HIV-1 pathogenesis and disease outcome (21–24). Evolution of HIV-1 toward highly pathogenic CXCR4-tropic variants has been previously linked to rapid disease progression (25, 26). We isolated virus from plasma samples as previously described by our group (27, 28) and determined virus coreceptor usage and replicative capacity pre- and post-LoH.

**FIG 3:**
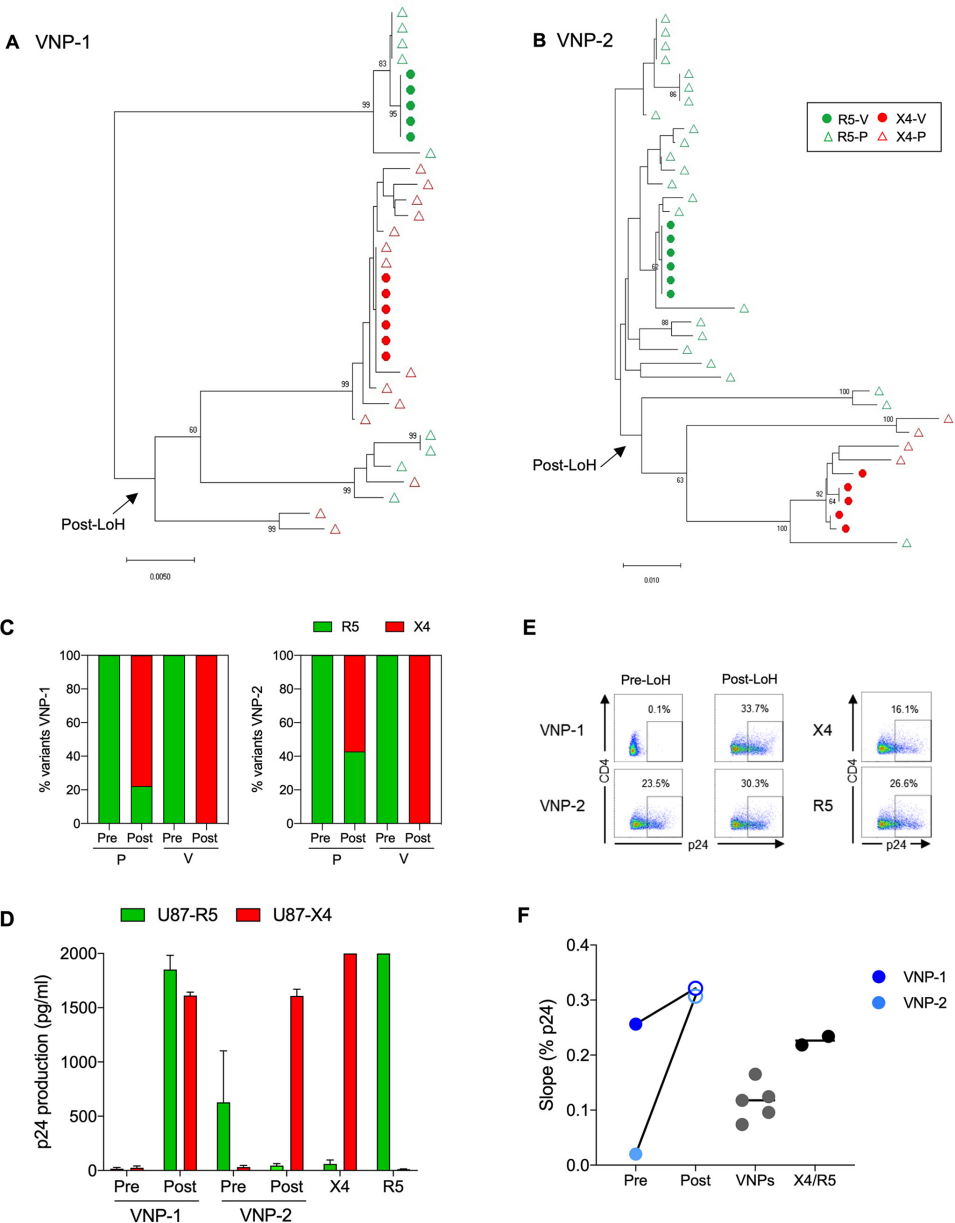
Viral coreceptor usage and replicative capacity in VNPs experiencing LoH. (A, B) Maximum likelihood phylogenetic tree of HIV-1 *env* clonal sequences obtained from pre- and post-LoH in plasma samples (P, triangles) and viral isolates (V, circles) are shown for VNP-1 (A) and VNP-2 (B). X4 sequences are indicated in red, and R5 sequences are in green. Branches indicate phylogenetic distance. Only bootstrap values greater than 60 are represented. An arrow indicate sequences at the LoH. (C) Percentage of clonal *env* sequences with R5 and X4 tropism from plasma (P) and viral isolates (V) inferred by PSSM and Geno2pheno predictive algorithms. (D) Phenotypic viral tropism experiments in U87-immortalized cell lines expressing CCR5 (U87-R5) or CXCR4 (U87-X4) infected with the pre- and post-LoH viral isolates from VNP-1 and VNP-2. Bal and NL43 were used as R5- and X4-tropic HIV-1 control viruses. The assays were performed in triplicate. Bars indicate the average p24 production. Error bars show standard deviation and error. (E) Viral replication was monitored by p24 production by flow cytometry up to 7 days postinfection. Representative flow cytometry plots are shown for intracellular p24 measurement at day 7 postinfection for VNP-1, VNP-2 isolates, and reference laboratory strains. Numbers in the quadrants indicate the percentage of cells. (F) Replicative capacity was calculated as the viral replication slope in the exponential growth phase for each viral isolate between pre-LoH (filled circle) and post-LoH (open circle). Isolates from VNPs not experiencing LoH and X4 and R5 laboratory strain were used for comparative purposes. P, plasma-derived *env* sequence; V, viral isolate-derived *env* sequence.

**FIG 4:**
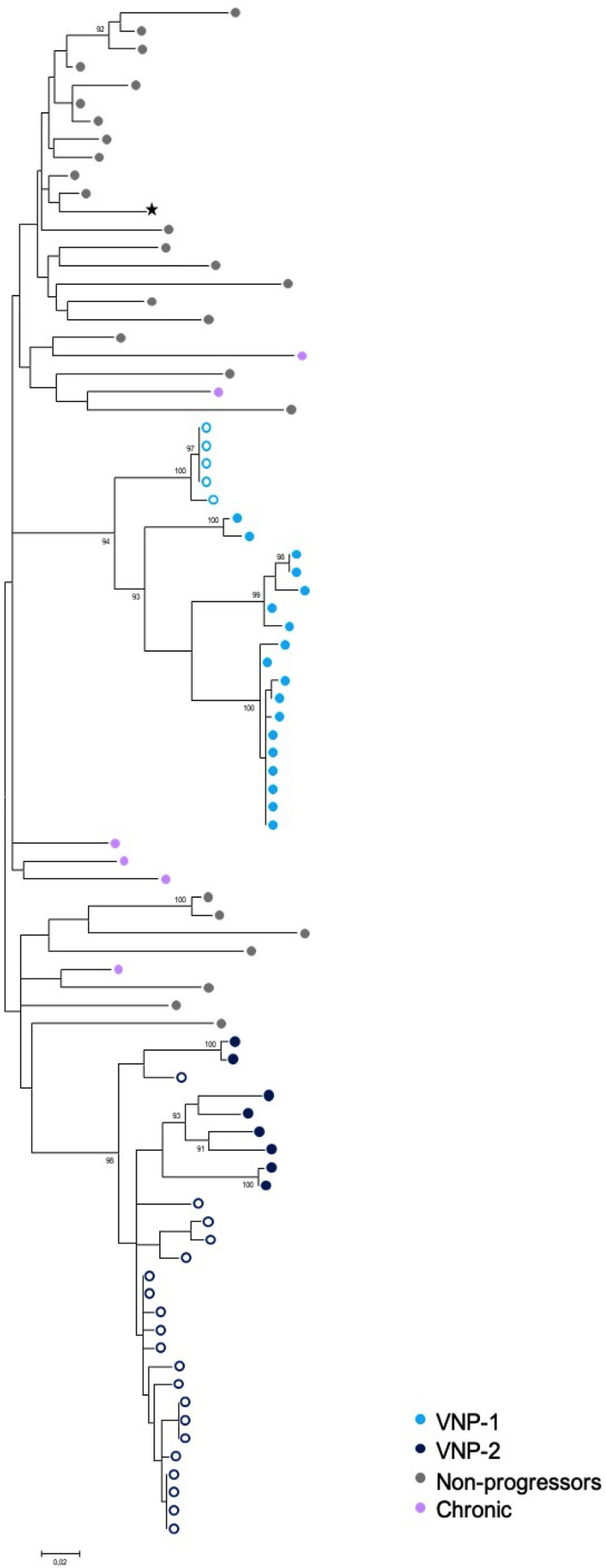
*Env* phylogenetic tree excluding evidence of HIV-1 superinfection events. Maximum likelihood phylogenetic tree of HIV-1 C2–V5 *env* clonal viral sequences obtained from pre-LoH (open circle) and post-LoH (filled circle) plasma and primary isolates are shown for VNP-1 (light blue) and VNP-2 (dark blue) subjects. *Env* sequences for HIV-1 B subtype from nonprogressors (gray), chronic HIV-negative individuals (purple), and a lab reference subtype B virus (89SP061, ★) were included in the analysis. Bootstrap values greater than 90% based on 1,000 bootstrap replicates are shown.

To evaluate changes in the coreceptor usage, we performed clonal phylogenetic analyses of the C2–V5 *env* region and phenotypic assays in pre- and post-LoH plasma samples (P) and viral isolates from plasma samples (V). As shown in Fig. 3A and B, clonal phylogenetic analyses of *env* revealed sequence evolution from CCR5 to CXCR4 usage in P and V sequences associated with LoH in both VNPs. Algorithm inference further demonstrated HIV-1 sequence evolution in P from 0 to 80% and from 0 to 60% of CXCR4 using virus at post-LoH in VNP-1 and VNP-2, respectively (Fig. 3C). Similarly, clonal sequences from V were 100% CXCR4 in both VNPs at post-LoH (Fig. 3C). Small discrepancies in the frequency of clonal CXCR4 virus between P and V samples may have revealed preferential outgrowth of CXCR4 viral isolates in *in vitro* culture. Phenotypic selection of CXCR4 virus upon the LoH was further confirmed in infectivity assays in U87-R5- or U87-X4-expressing cell lines as represented in Fig. 3D. Moreover, by using phylogenetic analyses of the C2–V5 *env* region, we confirmed virus relatedness between pre- and post-LoH samples in VNP-1 and VNP-2 and its circulating variants and excluded HIV-1 superinfection events as the cause of the LoH in VNPs (Fig. 4).

Limited and controversial information about the fitness of HIV-1 variants infecting VNPs is currently available (2, 29–31). Therefore, we determined whether differences in viral replication may contribute to LoH and associated with the changes in viral tropism. For 7 days, we monitored the kinetics of viral replication from the viral isolates in CD8-depleted peripheral blood mononuclear cell (PBMCs) by the expression of intracellular p24 protein (Fig. 3E). We observed a significant increase in viral replication of viral isolates in post-LoH coincident with the switch to CXCR4 coreceptor usage in both VNPs (Fig. 3F). This observation was particularly evident in the case of VNP-1, where the pre-LoH isolate showed impaired replication in culture and the post-LoH isolate showed a high replicative capacity. Overall, the replicative capacity of post-LoH viruses was high compared to viral isolates from unrelated VNPs that do not lose control and HIV-1 laboratory strains (Fig. 3F). These findings demonstrate evolution toward the selection of highly replicative CXCR4 HIV-1 variants associated with LoH in VNPs.

### Frequency of activated CD4^+^ and CD8^+^ T-cells and maturational phenotypes with LoH in VNPs.

Second, to evaluate the impact of cellular factors in LoH in VNPs, we determined the profile of T-cell activation and maturational phenotypes as characteristics potentially associated with nonpathogenic HIV-1 infection. Lower activation measured by the expression of HLA-DR and CD38 on CD4^+^ and CD8^+^ T-cells has been previously observed in VNPs and controllers (2, 3, 15). In addition, T-cell anergy in response to activation has been proposed to control HIV-1 pathogenesis in VNPs. Therefore, we measured changes in CD4^+^ and CD8^+^ T-cell activation and the T-cell response to polyclonal stimuli in pre- and post-LoH samples. Briefly, PBMCs were treated with phytohemagglutinin (PHA), concanavalin A (ConA), or anti-CD3/CD28 for 72 h and compared with unstimulated cells (basal conditions). We assessed CD4^+^ and CD8^+^ T-cell activation by HLA-DR and CD38 markers and maturational phenotypes by the combination of CD45RA, CCR7, and CD27 markers using flow cytometry.

**FIG 5:**
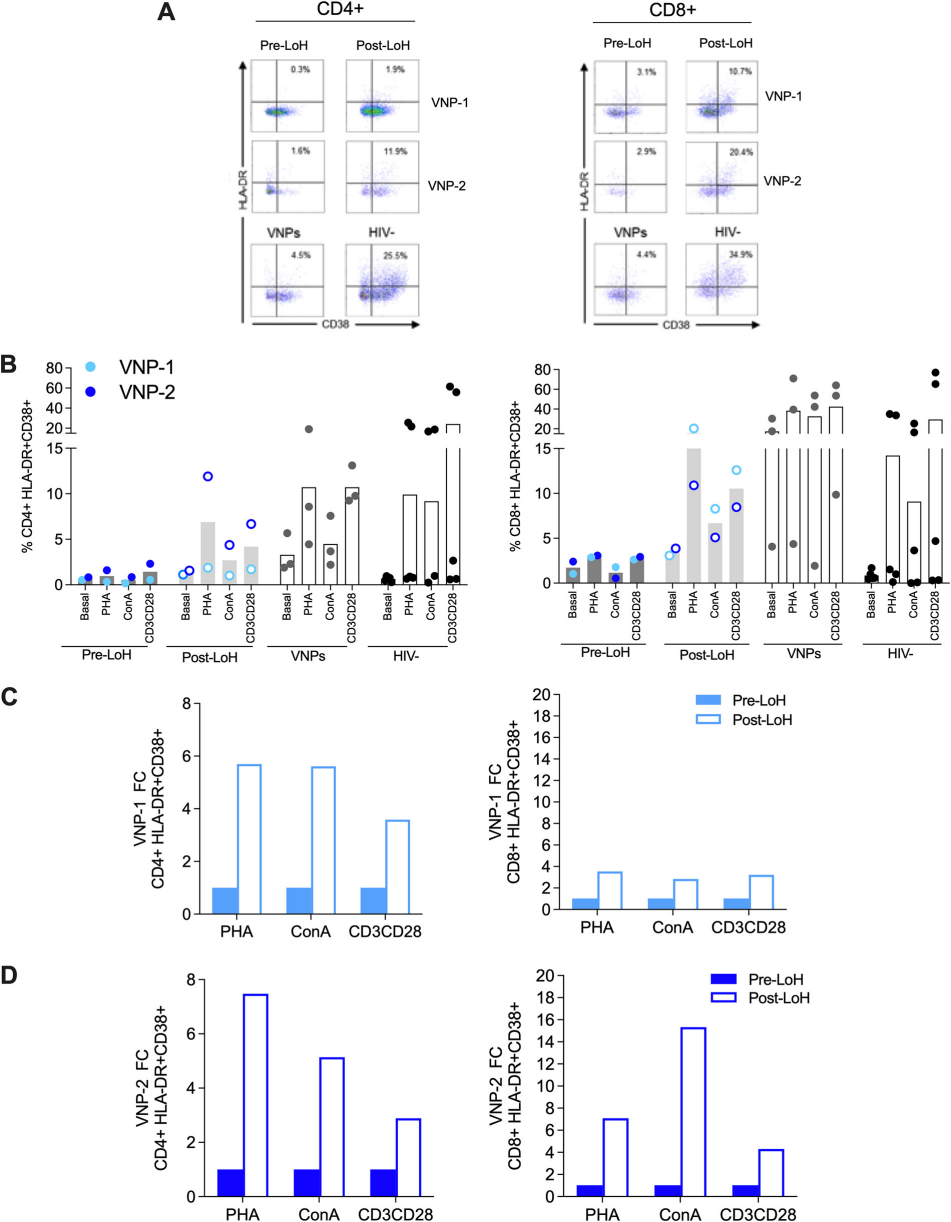
Activation of CD4^+^ and CD8^+^ T-cells in VNPs with LoH. CD4^+^ and CD8^+^ T-cell activation in VNP-1 and VNP-2 was monitored in PBCMs by the frequency of HLA-DR and CD38-positive cells, pre- and post-LoH under basal conditions and after 72 h of polyclonal stimulation with PHA, ConA, and anti-CD3/CD28. (A) Representative dot plots showing HLA-DR and CD38 expression in CD4^+^ and CD8^+^ T-cells 72 h post-PHA stimulation. Numbers in the quadrants indicate the percentage of cells. (B) Bar graphs represent the frequency of CD4^+^ HLA-DR+CD38^+^ (left) and CD8^+^ HLA-DR+CD38^+^ (right) T-cells in VNP-1 and VNP-2 under basal conditions and after stimulation. Pre-LoH (filled circles) and post-LoH (open circles) in VNP-1 and VNP-2, VNPs not experiencing LoH (VNPs, gray circles), and uninfected individuals (HIV−, black circles). (C, D) Bar graphs representing CD4^+^ and CD8^+^ T-cell activation as fold change (FC) of pre-LoH samples in relation with post-LoH in VNP-1 (C) and VNP-2 (D). PHA, phytohemagglutinin; ConA, concanavalin A.

Under basal conditions, we found a 2-fold increase in the frequency of T-cell activation in CD4^+^ and CD8^+^ cells measure by HLA-DR+CD38^+^ between pre- and post-LoH samples (Fig. 5B). Upon 72 h of stimuli, the observed difference in T-cell activation substantially increased in post-LoH samples but not pre-LoH samples for VNP-1 and VNP-2 (Fig. 3B). We noted high interindividual diversity in the response to polyclonal activation in VNPs and HIV-1 seronegative comparative groups, suggesting the presence of intrinsic host factors in the differential response to activation. Also, to monitor the variation in T-cell activation, we calculated the fold change (FC) as the ratio of the frequency of HLA-DR+CD38^+^ T-cells between pre- and post-LoH samples in CD4^+^ and CD8^+^ cells. We observed a 5.56-FC mean increase in the frequency of HLA-DR+CD38^+^ CD4^+^ and CD8^+^ T-cells upon activation at the LoH in VNPs (Fig. 5C and D). This augment was particularly marked in the case of CD8^+^ T-cells in VNP-2 reaching a 7.1- (PHA), 15.3- (ConA), and 4.3-FC (anti-CD3CD28) after polyclonal stimuli post-LoH (Fig. 5D). These data suggest the intrinsic changes in the T-cell response to activation at the LoH.

**FIG 6:**
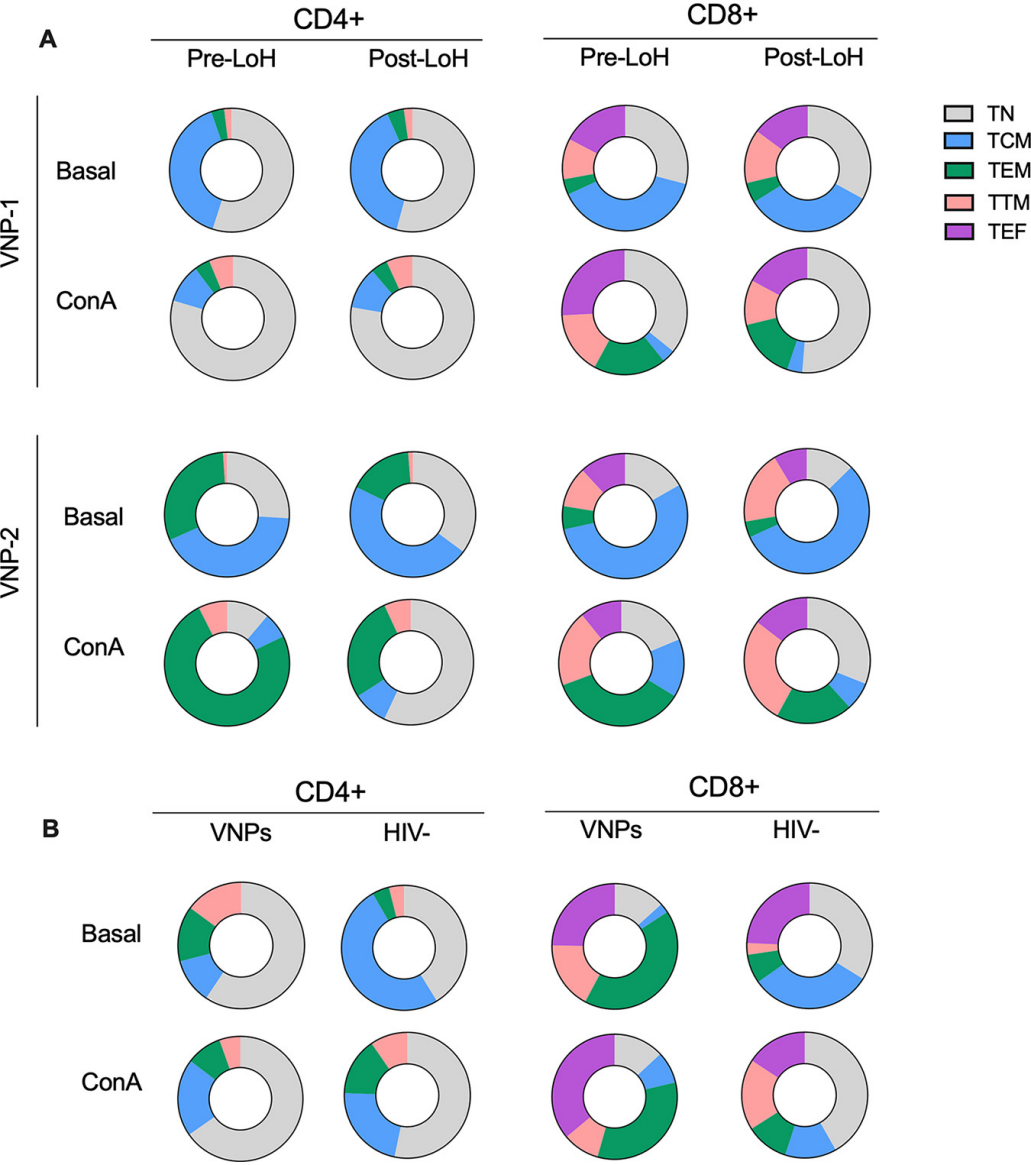
CD4^+^ and CD8^+^ T-cell subsets distribution in VNPs with LoH. PBMCs were stimulated for 72 h with PHA, ConA, or anti-CD3CD28 and stained with antibodies for CD4^+^ and CD8^+^ T-cell lineages (CD45RA, CCR7, CD27) and analyzed by flow cytometry to distinguish T-cell subsets: T naive (TN), T central memory (TCM), T effector memory (TEM), T transitional memory (TTM), and T effector (TEF) cells. (A) Donut charts show the frequency distribution pre- and post-LoH of CD4^+^ and CD8^+^ T-cell subsets in VNP-1 and VNP-2 under basal conditions and with ConA stimulation. (B) Donut charts show the median frequency distribution of CD4^+^ and CD8^+^ T-cell subsets in VNPs not experiencing LoH (*n* = 3) and in HIV-negative individuals (*n* = 5). Experiments did not include replicates as all subjects and data points are unique.

Moreover, we investigated potential changes in T-cell maturational phenotypes associated with LoH as described above. We observed conserved maturational phenotypes overtime in VNP-1 and VNP-2 in the absence of polyclonal activation (Basal). Maturational phenotypes were characterized by major contributions of TN and TCM in CD4^+^ and CD8^+^ T-cells despite LoH (Fig. 6A). Therefore, T-cell phenotypes determined upon polyclonal stimulation in our experimental setting were representative of the cellular response to stimulation. Overall, we found a general increase in the frequency of TN in CD4^+^ and CD8^+^ T-cells post-LoH upon ConA stimulation (Fig. 6A). We observed similar findings for the rest of the stimuli used (data not shown). In addition, there were particularities in the maturational phenotypes per individual. For VNP-1, the increase of TN cells in CD8^+^ T-cells was accompanied by an augment of TEM. For VNP-2, we found a preferential increase of TN and TEM cells in CD4^+^ and CD8^+^ T-cells post-LoH. Of note, there was a consistent reduction in the frequency of TEM upon stimulation in pre- and post-LoH samples in VNP-2 (74.1% versus 27.2% in CD4^+^ cells; 35.5% versus 19.6% in CD8^+^ cells) (Fig. 6A). Small alterations in T-cell maturational phenotype were found in VNPs that did not experience a loss of control and in healthy HIV-1-ve controls (Fig. 6B).

Taken together, these data suggest a contribution of alterations in the T-cell response to polyclonal activation identified by an increase in the frequency of HLA-DR+CD38^+^ CD4^+^ and CD8^+^ T-cells associated with LoH. However, the complexity of T-cell maturational phenotypes upon polyclonal stimulation in these two cases preclude a direct association between the increase of CD4^+^ and CD8^+^ activated T-cells with specific T-cell subsets.

### Stable and limited expression of CCR5 and CXCR6 in CD4^+^ T-cells in LoH VNPs.

Third, to determine the contribution of CCR5 and CXCR6 coreceptor expression in CD4^+^ T-cells as an additional host factor potentially involved in LoH, we assessed CCR5 and CXCR6 frequency in CD4^+^ T-cells upon polyclonal *in vitro* activation pre-LoH and post-LoH. Although the role of coreceptor expression in protection from infection in HIV-1 controllers and VNPs is still controversial (3, 15), polymorphisms in the CCR5 gene have been associated with HIV-1 outcome, and the deletion of 32 base-pairs in CCR5 results in protection from infection (32). Furthermore, polymorphism in the CXCR6 gene correlated with control in long-term nonprogressors (33). In VNPs, studies have supported the link between limited CCR5 expression on TCM and protection from infection in infants (15). However, other studies found similar expression of CCR5 on CD4^+^ T-cells subsets in adults (3). In addition, efficient use of the CXCR6 coreceptor has been shown in SIV infection as an alternative coreceptor for viral entry when CCR5 expression is low (34–36).

**FIG 7:**
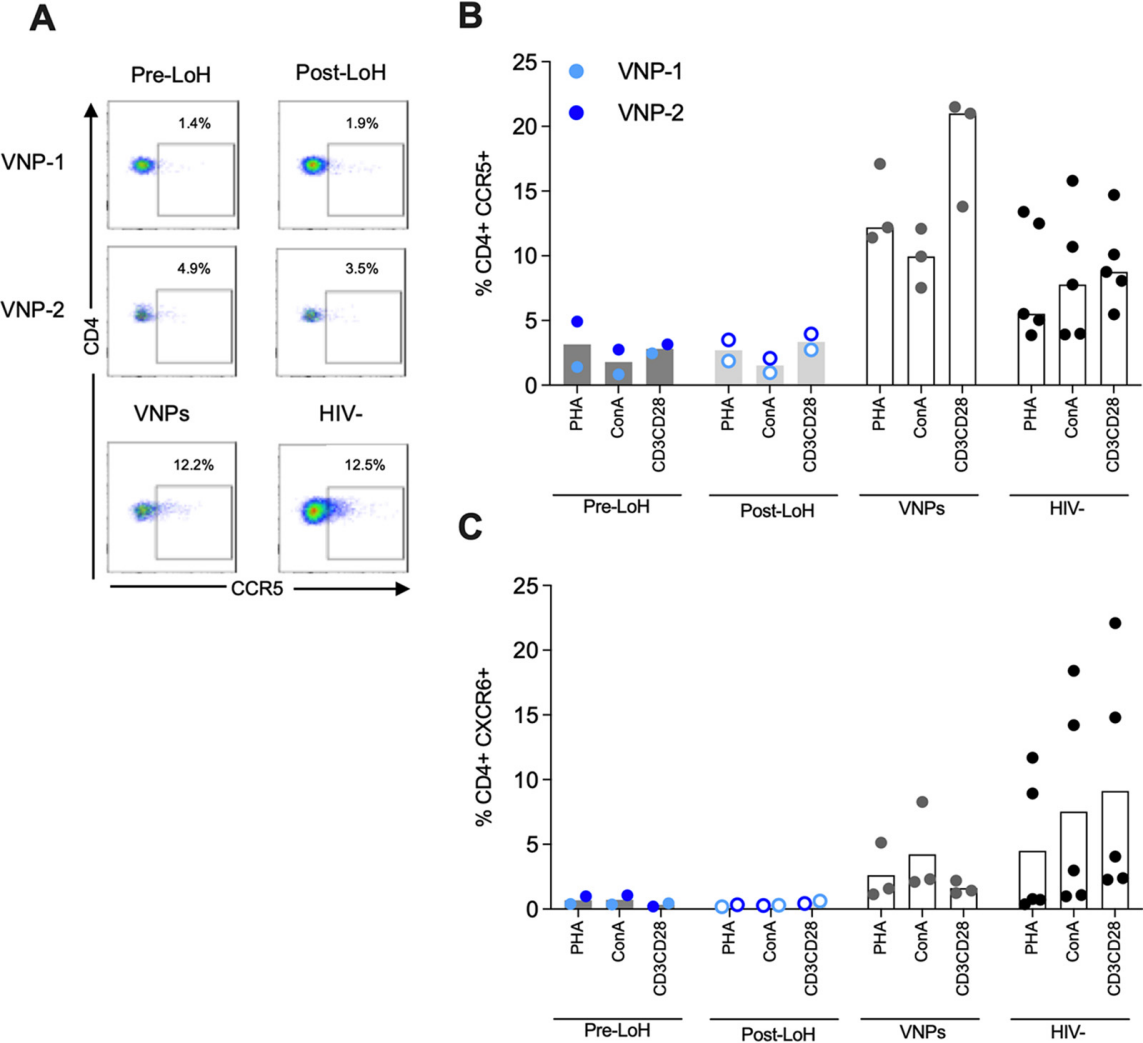
CCR5 and CXCR6 coreceptor expression on CD4^+^ T-cells in VNPs experiencing LoH. (A) Representative dot-plot of CCR5 expression. (B, C) Frequency of CD4^+^ expressing CCR5 (B) and CXCR6 (C) in pre-LoH (filled circle) and post-LoH (open circle) samples in VNP-1 and VNP-2. VNPs subjects not experiencing LoH and HIV-negative samples were included for comparative purposes. Bars represent median values. Experiments did not include replicates as all subjects and data points are unique.

We found a consistently low expression of CCR5 on CD4^+^ T-cells (<5%) independent of the polyclonal stimuli used (PHA, ConA, or anti-CD3/CD28) and pre-LoH and post-LoH sampling time in VNP-1 and VNP-2 (Fig. 7A). These results contrast with that of VNPs not experiencing LoH and seronegative individuals, where CCR5 expression was higher in all the conditions tested (median values were 12.20% for PHA, 9.95% for ConA, and 21% for anti-CD3CD28). We observed a similar pattern for CXCR6 expression on CD4^+^ T-cells. The expression of CXCR6 was scarce and stable upon stimuli and time points in CD4^+^ T-cells for VNP-1 and VNP-2 (<1%) (Fig. 7B). These data support stable and limited expression of CCR5 and CXCR6 coreceptors in CD4^+^ T-cells in VNPs with LoH.

## DISCUSSION

The phenomenon of loss of control has been reported in studies of natural HIV-1 controllers, including elite controllers (ECs) and long-term nonprogressors (LTNPs) (10, 11, 37–40) aiming to delineate the biological correlates of spontaneous HIV-1 control. Nonetheless, it is currently unknown whether viremic nonprogressors (VNPs), a very rare phenotype characterized by a paradoxically nonpathogenic HIV-1 infection and sustained CD4^+^ T-cells counts despite continuous viral load, may eventually lose CD4^+^ homeostasis (LoH). To our knowledge, this is the first report of LoH in VNPs. Our study is based on long-term clinical follow-up, biological sample availability, and the presence of comparative study groups focused on a deep characterization of viral and host factors in two case reports of LoH in VNPs.

In terms of viral factors, virus coreceptor usage and replicative capacity are essential determinants in HIV-1 pathogenesis and disease progression (28). Consistently, we found genotypic and phenotypic experimental evidence supporting HIV-1 evolution toward CXCR4 (X4) usage in both VNPs with LoH. The changes in coreceptor usage to R5X4 virus in VNP1- and X4 virus in VNP-2 were concomitant with an increase of virus replicative capacity and are a plausible explanation in LoH. This observation is particularly relevant in the case of VNP-2, where despite the initial genotypic and phenotypic characterization of R5 viruses in plasma and its isolation, the variants obtained did not grow in viral replication assays pre-LoH and contrast with the high replicative capacity of the X4 isolates post-LoH. These observations are in agreement with multiple reports by our group and others that associated changes in viral tropism from R5 to X4 with an increase in HIV-1 replication, pathogenesis, and progression to AIDS (25, 26, 29, 41, 42). Moreover, changes in HIV-1 coreceptor usage have been linked to tropism for specific CD4^+^ cellular subsets in the context of HIV-1 clade C strains. Thus, R5 clade C strains maintain a preference for infecting memory CD4^+^ T-cells during later stages of infection, whereas emergent X4 HIV-1 clade C strains preferentially target TN CD4^+^ T-cells (43). Although speculative, changes in CD4^+^ cellular subsets particularly associated with the homeostatic expansion of TN cells in VNPs may contribute to the selection of X4 variants with high replication over time.

In terms of host factors, nonpathogenic HIV-1 infection is dissociated from T-cell immune activation in VNPs mimicking the infection in sooty mangabeys, the African “natural” by SIV. T-cell immune activation is one of the hallmarks of HIV-1 disease progression (44). Consistent with the nonpathogenic model of SIV infection, a transcriptomic study showed lower immune activation in VNPs (1). However, there is still a discrepancy in the causality of the low immune activation status in VNPs (2, 3), and the potential anergy of T-cells to activation has been suggested in nonpathogenic HIV-1 infection. Thus, to rule out changes in the status of immune activation in VNPs with LoH, we assessed CD4^+^ and CD8^+^ T-cell activation and maturational phenotypes under basal conditions and response to TCR stimulation. Consistently, we observed an increase in the frequency of activated CD4^+^ and CD8^+^ T cells with LoH both in the absence of stimuli and upon polyclonal stimulation (>5-FC increase). Together with the increase in the frequency of activated T cells in the context of polyclonal activation, we traced a general increase in naive CD4^+^ and CD8^+^ T-cells post-LoH. This observation is in line with an interleukin-7 (IL-7) dependence expansion of TN cells to maintain CD4^+^ T-cell homeostasis in VNPs as recently reported (8). Our results may be consistent with potential alterations in the responsiveness to activation by T cells proposed and changes in the maturational phenotype in VNPs as contributing factors to LoH.

Furthermore, we assessed CCR5 and CXCR6 expression in CD4^+^ T-cells as additional host factors potentially influencing LoH in VNPs. Deletion of 32 bp in the CCR5 gene protects from HIV-1 acquisition or disease progression (32). Similarly, polymorphisms in CXCR6 have been associated with viral control in long-term nonprogressors (33). Also, low expression of CXCR6 and CCR5 has been found in elite controllers (45, 46). We identify very low and stable levels of both CCR5 and CXCR6 expression on CD4^+^ T-cells despite LoH in VNPs. The level of expression was below the median expression found in VNPs not experiencing LoH or healthy donors, indicating that low expression of CCR5 and CXCR6 is not a common feature among VNPs and suggesting diversity within the VNPs phenotype. These observations are in agreement with the low expression of CCR5 found in central memory CD4^+^ in HIV-1 pediatric VNPs and SIV infection to sooty mangabeys (7, 15). Limited and stable expression of CCR5 and CXCR6 is a plausible mechanism associated with the VNPs phenotype by reducing CD4^+^ target availability in the study cases driving viral evolution over time toward X4 variants as described here.

Despite the presence of protective HLA alleles in VNP-1 and VNP-2, the identified presence of CD8^+^ escape mutations in immunodominant targeted regions pre-LoH points to a limited role of CD8^+^ T-cell immunity in these two cases (19, 20). Moreover, continuous viral replication in VNPs separates them from EC, where the contribution of protective HLA alleles and CD8^+^ T-cell immunity in viral control is established.

Our results present some limitations, including the number of study subjects and sample availability. These limitations preclude establishing consistent longitudinal data on T-cell activation and maturational phenotypes before LoH and direct causality between the study factors and the phenotype. Therefore, further studies will be necessary to corroborate our findings on T-cell activation profiles and maturational status together with additional factors. Moreover, given that the VNP phenotype is more prevalent in children than adults (15, 47), especially in Africa and Southeast Asia, it would be of great interest to investigate to what extent similar observations extend to pediatric VNPs.

In summary, the present study reports for the first time two cases of LoH in VNPs. Our findings suggest that LoH in VNPs results from viral evolution toward highly pathogenic X4 strains potentially led by bottleneck events due to very limited and stable expression of CCR5 and CXCR6 in CD4^+^ T-cells over time. These data bring novel insights into the correlates of nonpathogenic infection pointing at therapeutics limiting CCR5 and CXCR6 expression to control HIV-1 pathogenesis. However, our findings raise awareness of the potential of viral evolution to overcome these strategies in the long term.

## MATERIALS AND METHODS

### Study subjects and samples.

HIV-1 viremic nonprogressors (VNPs) were defined as cART-naive HIV-1-infected individuals whose median absolute CD4^+^ T-cell counts was >500 cells/mm^3^, with a plasma HIV-1 RNA load of >10^4^ copies/ml and without clinical symptoms for more than 8 years of follow-up. The transition to LoH was defined as a consecutive rise in viral load (>0.5 log_10_ VL) and/or a decrease of 30% CD4^+^ T-cell counts within 1 year. Based on these criteria, we identified two VNPs among IrsiCaixa’s VNP cohort, termed VNP-1 and VNP-2, who experienced disease progression. Clinical and virological characteristics are summarized in Table 1. Biological samples from both VNP subjects were available pre- and post-LoH. High-resolution HLA class I typing was performed by sequence-based typing methods. In addition, three VNPs from IrsiCaixa’s VNP cohort and five seronegative donors from the Catalan Blood and Tissue Bank were included for comparison purposes. Of note, VNP-1 and VNP-2 were both males and of the same age. Regarding the VNPs that did not experience loss of control, two individuals were male and one was female, all aged matched. The information regarding age and sex of the HIV-negative individuals was not available because this information was not provided for the blood samples obtained from the Catalan Blood and Tissue Bank.

### Ethics statement.

Written informed consent was obtained from all study participants (IrsiCaixa’s VNPs Cohort and healthy donors), and the study followed all bioethical and legal requirements. The study was approved by the Ethics Committee of the Hospital Universitari Germans Trias i Pujol (ethics committee approval number IVAN EO-12-017). All clinical investigations were conducted according to the standards indicated by the Declaration of Helsinki.

### Virus isolation from plasma samples and PBMCs.

HIV-1 primary isolates from retrospective pre- and post-LoH plasma samples of the two VNP individuals were obtained and further propagated and titrated as previously described (28, 48). Briefly, viruses were isolated from cryopreserved plasma samples by using anti-CD44, according to the manufacturer’s instructions (Miltenyi Biotech, Cologne, Germany). Also, pooled CD8-depleted peripheral blood mononuclear cells (PBMCs) from healthy donors were stimulated under three different conditions (3 × 3 Method; Miltenyi Biotech, Cologne, Germany): low-dose phytohemagglutinin (PHA; 0.5 μg/ml; Sigma-Aldrich, St. Louis, MO), high-dose (PHA 5 μg/ml; Sigma-Aldrich, St. Louis, MO), or late-bound anti-human CD3 monoclonal antibody functional grade (OKT3 10 μl/mL; eBiosciences, San Diego, CA) at 37°C. Seventy-two hours later, cells from the three different conditions were pooled, and 3 × 10^6^ cells/ml were infected with 200 μl of extracted virus for 2 to 4 h at 37°C. After infection, the cells were resuspended to a final concentration of 10^6^ cells/ml in RPMI 1640 medium (Invitrogen, Thermo Fisher Scientific, Waltham, MA) plus 10% fetal calf serum (FCS; Gibco, Thermo Fisher Scientific, Waltham, MA) and penicillin/streptomycin, supplemented with 100 U of interleukin-2 (IL-2)/ml (Roche, Basel, Switzerland). The cultures were fed weekly with 10^6^ cells/ml freshly 3 × 3-stimulated CD8-depleted PBMCs, and viral growth was monitored by p24 enzyme-linked immunosorbent assay (ELISA; Perkin Elmer, Madrid, Spain) twice a week. Viral stocks were harvested when p24 was >10^4^ pg/ml, filtered through a 0.45-μm syringe filter (Merck), and stored at −80°C until use.

### Viral titration and tropism assays.

The 50% tissue culture infective dose (TCID_50_) of each viral isolate from VNP individuals was determined in TZM-bl using the Reed and Muench method, as previously described (49). Briefly, freshly trypsinized TZM-bl cells (National Institutes of Health [NIH] AIDS Reagent Program, catalog number 8129) were incubated on a 96-well plate for 48 h at 37°C. Then, serially diluted viral isolates were added to the culture and incubated for 5–7 days. Following the incubation period, 100 μl of culture medium was removed from each well and replaced by a luciferase reporter gene assay system reagent (Bright-Glo™ luciferase assay system, Promega, Madison, WI). After a short incubation (minimum of 2 min) in agitation at room temperature, lysates were measured in a luminometer (Perkin Elmer EnSight, Madrid, Spain).

Viral tropism was measured in U87-immortalized cell lines expressing CCR5 or CXCR4 coreceptors (NIH AIDS Reagent Program, catalog numbers 4035 and 4036, respectively) as previously described (28, 50, 51). In a 96-well plate, 5,000 cells were seeded and infected with 4,000 pg of p24 for each viral isolate in three independent biological replicates. The HIV-1_NL4-3_ (X4-tropic virus) and HIV-1_Bal_ (R5-tropic virus) HIV-1 laboratory strains were used as positive controls. After 24 h, the viruses were washed three times with phosphate-buffered saline (PBS), and fresh medium was added to a final volume of 200 μl. The presence of syncytia was determined 5 days postinfection by nuclear DAPI staining (50 μg/ml; Roche, Basel, Switzerland), and viral growth was measured by p24 in the supernatant by ELISA (Perkin Elmer, Barcelona, Spain). Viral isolates were considered positive for a particular coreceptor usage by the presence of syncytia under the microscope and by p24 quantification over 300 pg/ml.

### Viral sequencing and phylogenetic analysis.

The Gag-protease region of the HIV-1 genome from the viral isolates obtained was sequenced, and phylogenetic analyses were performed as previously described (52). HIV-1 viral subtypes were determined by phylogenetic analysis of the Gag-protease region of the viral isolates, and subtype-specific consensus sequences were obtained from Los Alamos Database. Also, virus subtype was determined using the REGA subtyping tool version 3.0 (http://dbpartners.stanford.edu:8080/RegaSubtyping/stanford-hiv/typingtool/).

The clonal sequences of the *env* region were obtained from pre- and post-LoH viral isolates and plasma samples. Briefly, viral RNA was extracted and amplified by limiting dilution using the OneStep reverse transcription (RT)-PCR kit (Qiagen, Valencia, CA), followed by a nested PCR using Phusion High Fidelity (Thermo Fisher Scientific, Waltham, MA) with specific primers for the C2–V5 region, as previously described (10). Nucleotide sequences were determined with the Big Dye terminator cycle sequencing kit (Applied Biosystems, Thermo Fisher Scientific, Waltham, MA) in an ABI 3730 sequencer (Applied Biosystems). Nucleotide sequences were assembled using the SeqMan program (DNASTAR) and aligned by the MEGA program (v6.0). Maximum likelihood trees with 1,000 bootstrap replicates were performed with the MEGA program (v6.0). Initial tree(s) for the heuristic search were obtained by applying the neighbor-joining method to a matrix of pairwise distances estimated using the maximum composite likelihood (MCL). The Web Position Specific Scoring Matrix (PSSM, https://indra.mullins.microbiol.washington.edu/webpssm/) and Geno2pheno (g2p, https://coreceptor.geno2pheno.org/) predictive algorithms were used to infer the coreceptor usage of the *env* clones based on the V3 loop sequences (53, 54).

### Replicative capacity assays.

Following the 3 × 3 method, 6 × 10^6^ PHA (1 μg/ml)-stimulated CD8-depleted fresh PBMCs pooled from three seronegative individuals were infected with the VNP-1 and VNP-2 viral isolates and also control virus to a multiplicity of infection (MOI) of 0.01 for 4 h at 37°C in three independent biological replicates. After infection, the cells were washed three times with 1× PBS and resuspended to a final concentration of 10^6^ cells/ml in R10 medium (RPMI 1640 medium supplemented with 10% FBS and 100 μg/ml penicillin-streptomycin) supplemented with IL-2 (50 U/ml). A total of 200 μl of culture were collected at every time point and replaced with 200 μl of fresh medium. Viral growth was monitored by intracellular p24 staining by multiparametric flow cytometry at days 1, 4, and 7 postinfection. Viral supernatants were collected and sequenced to confirm virus identity and the lack of cross-contamination.

### T-cell activation *in vitro* assays.

Cryopreserved PBMCs from the two VNP patients pre- and post-LoH, as well as from three VNPs not experiencing LoH and five seronegative individuals were thawed and rested in R10 medium for 4 to 6 h at 37°C before stimulation. Then, the cells were supplemented with IL-2 (50 U/ml) and cultured under different stimuli conditions: PHA (2 μg/ml, Sigma-Aldrich, St. Louis, MO), concanavalin A (ConA; 1 μg/mL, Sigma-Aldrich, St. Louis, MO), or anti-CD3/CD28 (1 μg/ml, BD Biosciences, San Jose, CA). To evaluate the activation status, PBMCs were analyzed by light microscopy to confirm cellular aggregation and refractivity 72 h poststimulation. At 0 and 72 h postactivation, 500,000 cells were collected and stained for viability, CD4^+^ and CD8^+^ T-cell lineage (CD3, CD4, CD8, CD45RA, CCR7, CD27), activation (HLA-DR and CD38), and coreceptor (CCR5 and CXCR6) expression. For comparative purposes, the total percentage of activation levels for each condition was normalized to 10. Viability was measured upon PBMCs thawing by flow cytometry in all samples. A similar number of viable cells were included per sample and per experimental condition. The viability of samples tested was 75.50% pre-LoH and 89.80% post-LoH for VNP-1 and 70.5% pre-LoH and 90.8% post-LoH for VNP-2. Viability in VNPs and HIV-negative comparative samples tested was >70%. The sampling time point for VNPs was similar to post-LoH samples, and the sampling time point for HIV-negative samples matches the time of the experiments.

### Flow cytometry analysis.

For the T-cell activation assays, PBMCs were used for multiparametric flow cytometry analysis of cell surface staining. Briefly, the cells were collected at 0 and 72 h postactivation, washed with 1× PBS, and stained with a Live/Dead probe (APC-Cy7, Invitrogen, Thermo Fisher Scientific, Waltham, MA) for 25 min in the dark. Next, the cells were washed twice with 1× PBS and surface stained for 25 min in the dark with CD3 (Alexa Fluor 700, BD Biosciences, San Jose, CA), CD4 (Alexa Fluor-647, BD Biosciences, San Jose, CA), CD8 (BV650, BD Biosciences, San Jose, CA), CCR5 (PE-Cy7, BD Biosciences, San Jose, CA), and CXCR6 (BV421, BD Biosciences, San Jose, CA) markers of coreceptor expression and CD38 (BV711, BD Biosciences, San Jose, CA) and HLA-DR (PerCP-Cy5.5, BD Biosciences, San Jose, CA) immune activation markers. The CD4^+^ and CD8^+^ T-cell subsets were differentiated on the basis of CD27 (BV605, BD Biosciences, San Jose, CA), CD45RA (BV786, BD Biosciences, San Jose, CA), and CCR7 (PE-CF594, BD Biosciences, San Jose, CA) expression: naive (TN: CD45RA^+^, CD27^+^, CCR7^+^), effector (TEF: CD45RA^+^, CD27^−^, CCR7^−^), central memory (TCM: CD45RA^−^, CD27^+^, CCR7^+^), transitional memory (TTM: CD45RA^−^, CD27^+^, CCR7^−^), and effector memory (TEM: CD45RA^−^, CD27^−^, CCR7^−^), as previously described (3, 55). Stained samples were washed twice with 1× PBS and resuspended in 1% formaldehyde (Sigma-Aldrich, St. Louis, MO) before the acquisition. Flow cytometry acquisition was performed on a Fortessa flow cytometer (BD Biosciences) at the flow cytometry core facility at the Germans Trias i Pujol Research Institute within 12 h of staining. Data analysis was performed using FlowJo software version 10.0.7 (Tree Star, Ashland, OR).

To monitor viral replicative capacity, we performed HIV-1 p24 intracellular staining. Briefly, after surface staining with a Live/Dead probe (APC-Cy7, Invitrogen) and the CD4 antibody (Alexa Fluor-647, BD Biosciences), the cells were washed twice in 1× PBS, fixed, and permeabilized (Fix & Perm cell fixation cell permeabilization kit, Thermo Fisher Scientific) and washed twice again in 1× PBS. Finally, the cells were stained with the p24 antibody KC.57-PE (Beckman Coulter) for 20 min in the dark, washed two additional times with 1× PBS, and fixed in 1% formaldehyde solution (FA; Sigma-Aldrich, St. Louis, MO) to be acquired on LSRII flow cytometer (BD Biosciences).

### Statistical analyses.

The analysis focuses on the calculation and representation of relevant descriptive data rather than the usual hypothesis tests. In the analysis of T-cell activation and maturational phenotypes, the data show median values within maturational phenotypes normalized to 100%. All data analyses were performed using GraphPad Prism, version 7.04 (GraphPad Software, Inc., San Diego, CA).
